# Making Workshops Work: Insights from EDAMAME

**DOI:** 10.1128/mSystems.00467-19

**Published:** 2019-08-20

**Authors:** Jaclyn N. Taroni

**Affiliations:** a Childhood Cancer Data Lab, Alex’s Lemonade Stand Foundation, Philadelphia, Pennsylvania, USA

**Keywords:** education, training

## Abstract

Microbiology, like many areas of life science research, is increasingly data-intensive. As such, bioinformatics and data science skills have become essential to leverage microbiome sequencing data for discovery. Short intensive courses have sprung up as formal computational training opportunities at individual institutions fail to meet demands. In this issue, Shade et al. (A. Shade, T. K. Dunivin, J. Choi, T. K. Teal, et al., mSystems 4:e00297-19, 2019, https://doi.org/10.1128/mSystems.00297-19) share their experience and approach in executing the annual, weeklong Explorations in Data Analysis for Metagenomic Advances in Microbial Ecology (EDAMAME) workshop from 2014 to 2018. EDAMAME introduced learners to general scientific computing concepts and domain-specific data analysis approaches. Workshop learners self-reported appreciable gains in understanding and ability. This report on the EDAMAME workshop strategy and lessons learned will help others in the life sciences to plan, execute, and assess short hands-on computing-intensive courses that support research in a particular domain.

## Abstract (precis)

Microbiology, like many areas of life science research, is increasingly data-intensive. As such, bioinformatics and data science skills have become essential to leverage microbiome sequencing data for discovery. Short intensive courses have sprung up as formal computational training opportunities at individual institutions fail to meet demands. In this issue, Shade et al. (A. Shade, T. K. Dunivin, J. Choi, T. K.

## COMMENTARY

The practice of life science research is no longer separate from scientific computing. The gap between the needs for bioinformatics expertise and the bioinformatics expertise in the biomedical workforce has been noted since the 1990s, but formal training opportunities have not kept up with demand (1, 2). To conduct rigorous, reproducible computing-intensive research requires two sets of skills: scientific computing basics (e.g., coding, version control) (3, 4) and an understanding of approaches, tools, and pitfalls specific to one’s domain. The highly rated, weeklong Explorations in Data Analysis for Metagenomic Advances in Microbial Ecology (EDAMAME) workshop—97% of learners in the 5-year time span rated the course as “good” or “very good” (5)—taught microbiome researchers both sets. Shade and colleagues report their objectives, strategies, and lessons learned in administering EDAMAME and give readers a deeper insight into what it takes to make short courses work.

A weeklong intensive format is attractive: short enough for researchers from various career stages to attend (2), but long enough to impart some best practices. The focus of EDAMAME, and of workshops like it, is on learners developing computational skills and confidence through hands-on experience. Participants in short-format training are more likely to be motivated if they have a particular problem they would like to solve or, as noted by Shade et al. (5), if they have struggled with analysis in the past. The specificity of EDAMAME, in contrast to both more-general “bioinformatics boot camps” and introductions to programming languages such as R, likely contributes to effectiveness and to creating a sense of community. Emphasis on specific skills and tooling necessitates that workshop material keeps pace with both participant needs and new methodologies. Short courses are likely better suited to adapt to rapidly changing technologies than traditional semester-long instruction. A less apparent advantage of this structure is the ability to maximize the impact of training by including learners who may not have the same opportunities at their home institution and international learners in particular (5).

The demand for training in computational approaches makes the focused, intensive short-course format an appealing solution for closing the skill gap in a given community. However, the challenges and costs associated with the design and execution of workshops are often unclear to stakeholders. It is not enough for workshop instructors to have subject matter expertise (6) and physical space. Even before diving into frequent deeper issues such as how to assess if learning is occurring (7), a number of questions arise. What is the “right” length for a workshop? How many instructors are needed? What resources should be used for computing, and what are the logistical challenges associated with them? And, importantly, how much will this cost? The multiyear perspective provided by Shade et al. is a valuable resource for those organizing computing- and data-intensive workshops within and outside the microbiology community, in regard to both these seemingly simple questions and issues such as course structure and assessment.

There are less obvious requirements for running an effective workshop with an outsized impact. The EDAMAME course was held annually and supported 23 to 26 learners in each session. Shade et al. aimed to extend the reach of this relatively low-throughput model by targeting the learners who were most likely to benefit from the workshop and to go on to share the experience. Their commitment to promoting diversity required thoughtfulness in advertising to learners, selecting participants, and creating a friendly and welcoming learning environment. In the spirit of Software and Data Carpentries (www.carpentries.org), the EDAMAME workshop materials are openly shared on GitHub, which greatly enhances the benefit of the material (8). All the while, crafting a workshop may be viewed as less valuable than other scholarly activities in the absence of external funding (5). I strongly encourage those organizing similar workshops to take note of these factors as well.

Success in designing and delivering a successful bioinformatics workshop is no small feat. The value of sharing experiences and perspectives among instructors teaching short bioinformatics courses has been noted in national programs (7) and is highlighted by the founding of the Life Science Trainers community (https://lifescitrainers.org/). Shade et al. generously walk readers through the workshop process from the inception of the EDAMAME workshop to what came next. Perhaps the most exciting section of the article is the description of the plans for the workshop’s future—how EDAMAME is being adapted to accommodate more learners in different settings. Discussions like these will be essential to building life scientists’ computing skills going forward, as the demand has not yet shown signs of slowing down.

## References

[1] MacLean M, Miles C 1999 Swift action needed to close the skills gap in bioinformatics. Nature 401:10. doi:10.1038/43269. 10485694

[2] Attwood TK, Blackford S, Brazas MD, Davies A, Schneider MV 2019 A global perspective on evolving bioinformatics and data science training needs. Brief Bioinform 20:398–404. doi:10.1093/bib/bbx100. 28968751 PMC6433731

[3] Lowndes JSS, Best BD, Scarborough C, Afflerbach JC, Frazier MR, O’Hara CC, Jiang N, Halpern BS 2017 Our path to better science in less time using open data science tools. Nat Ecol Evol 1:0160. doi:10.1038/s41559-017-0160. 28812630

[4] Wilson G, Bryan J, Cranston K, Kitzes J, Nederbragt L, Teal TK 2017 Good enough practices in scientific computing. PLoS Comput Biol 13:e1005510. doi:10.1371/journal.pcbi.1005510. 28640806 PMC5480810

[5] Shade A, Dunivin TK, Choi J, Teal TK, Howe AC 2019 Strategies for building computing skills to support microbiome analysis: a 5-year perspective from the EDAMAME workshop. mSystems 4:e00297-19. doi:10.1128/mSystems.00467-19. PMC6702294 31431509

[6] Tractenberg RE 10 8 2018, posting date. Achieving sustainability and transfer with short term learning experiences. SocArXiv doi:10.31235/osf.io/jsfe9.

[7] McGrath A, Champ K, Shang CA, van Dam E, Brooksbank C, Morgan SL 2019 From trainees to trainers to instructors: sustainably building a national capacity in bioinformatics training. PLoS Comput Biol 15:e1006923. doi:10.1371/journal.pcbi.1006923. 31246949 PMC6597034

[8] Wilson G 2014 Software Carpentry: lessons learned. F1000Res 3:62. doi:10.12688/f1000research.3-62.v2. 24715981 PMC3976103

